# Workability and Mechanical Properties of PVA Fiber-Reinforced Concrete with Hybrid Dune Sand and Steel Slag Aggregates

**DOI:** 10.3390/ma18132956

**Published:** 2025-06-22

**Authors:** Yanhua Liu, Xirui Wang, Senyan Jiang, Qingxin Ren, Tong Li

**Affiliations:** 1 School of Civil Engineering and Transportation, Foshan University, Foshan 528225, China; liuyanhua@fosu.edu.cn (Y.L.); renqingxin@fosu.edu.cn (Q.R.); 2 College of Water Conservancy, Shenyang Agricultural University, Shenyang 110866, China; 18740048284@163.com (X.W.); jsy573@163.com (S.J.)

**Keywords:** dune sand, steel slag, PVA fiber-reinforced, workability, mechanical properties

## Abstract

To mitigate ecological damage from excessive natural aggregate extraction, this study developed an eco-friendly concrete using dune sand and steel slag as natural aggregates, enhanced with polyvinyl alcohol (PVA) fibers. Through orthogonal testing, the effects of the dune sand replacement ratio, steel slag replacement ratio, PVA fiber length, and PVA fiber content on concrete workability and mechanical properties were analyzed. The results show that slump exceeded 120 mm (meeting engineering requirements) in mixes except that with 40% dune sand, 60% steel slag, 18 mm PVA fiber length, and 0.4% PVA fiber content; 50% steel slag replacement significantly improved mechanical properties, yielding a 21.2% increase in 28 d compressive strength when replacement increased from 30% to 50%; 20% dune sand replacement for river sand is optimal; and while increased PVA content enhanced splitting tensile and flexural strengths, both its length and content should not exceed 9 mm and 0.3%, respectively. The concrete delivers acceptable performance while providing dual environmental benefits: reduced aggregate consumption pressure and achieved high-value-added dune sand–steel slag utilization.

## 1. Introduction

At present, river sand is mainly used as the fine aggregate of concrete. As one naturally consumes unrenewable resources, the massive exploitation of river sand severely destroys natural resources and makes the cost of river sand resources increase. It has been reported that the demand for natural sand increased approximately 23-fold from 1900 to 2006 [1], while its consumption has doubled over the past two decades [2]. This excessive depletion of natural resources has led to serious environmental degradation over time [3]. Therefore, it is urgent to explore new alternative fine aggregates. Now the desert covers about 18% of Chinese land area, and dune sand resources are relatively abundant [4]. Dune sand has been demonstrated as a viable alternative aggregate for concrete [5].

In recent years, steel slag has occupied substantial land resources, and waste liquids from steel slag have penetrated surrounding soil, causing environmental pollution. Steel slag is an underutilized industrial byproduct, with less than 25% being recycled in China despite its generation in steel production processes [6]; consequently, most steel slag is still landfilled as industrial waste. Research on utilizing waste steel slag as coarse aggregate in concrete holds significant environmental importance.

In addition, for concrete members, which are limited by their maximum crack width, their design bearing capacity fails to reach the ideal state and cannot fully utilize the advantages of concrete [7]. Some scholars [8,9,10] believe that concrete cracks can be reduced by adding polyvinyl alcohol (PVA) fibers to concrete, leveraging the hydrophilicity of PVA fibers to lower the evaporation rate. In fact, PVA fibers themselves can absorb energy, improve the mechanical properties of concrete, and inhibit crack development. Notably, free calcium oxide (f-CaO) in steel slag aggregates compromises volumetric stability [11]. Moreover, incorporating fine-grained dune sand exacerbates drying shrinkage of concrete [12]. PVA fiber incorporation simultaneously counteracts both detrimental effects: the hydrophilic properties suppress f-CaO hydration expansion [13], while its fiber-bridging effect compensates for shrinkage stresses [14].

Under these circumstances, researchers have conducted studies on dune sand concrete, steel slag concrete, and PVA fiber-reinforced concrete. Research on dune sand concrete has been conducted by researchers [15,16,17,18,19,20]. These studies focused on workability, strength, fluidity, plastic viscosity, and frost resistance of concrete. The results demonstrate that dune sand significantly affects concrete performance and improves frost resistance. In addition, studies by Felekoglu et al. [21], Qasrawi et al. [22,23], and He et al. [24] demonstrated that adding an appropriate amount of steel slag to concrete utilizes waste effectively and enhances workability, flexural strength, compressive strength, and splitting tensile strength. Recently, Alhozaimy et al. [25], Noushini et al. [26], Lin [27], and Qian et al. [28] have explored the influence of PVA fibers on concrete properties. Their results indicate that impact resistance, flexural strength, and flexural strength improve with optimal PVA fiber lengths and mix proportions.

Beyond single-factor studies, researchers have investigated the combined effects of dune sand, steel slag, and PVA fibers on concrete strength and workability over many years [29,30,31,32,33]. However, existing studies predominantly focus on substituting either coarse or fine natural aggregates with alternative materials, whereas research involving simultaneous replacement of both remains scarce. Therefore, in the current study, steel slag replaces natural gravel and dune sand substitutes natural sand to prepare environmentally friendly concrete, with PVA fiber added to enhance its performance.

## 2. Experiment

### 2.1. Materials

This study employed ordinary Portland cement (OPC, P⋅O 42.5) from LNJD Cement Factory, with properties detailed in Table 1. Two coarse aggregates (natural pebble and steel slag) were utilized; their physical properties are presented in Table 2. Prior to utilization, the steel slag was stockpiled at a landfill site for two years. This duration is sufficient to neutralize potential stability risks from f-CaO content [34].

**Figure 1:**
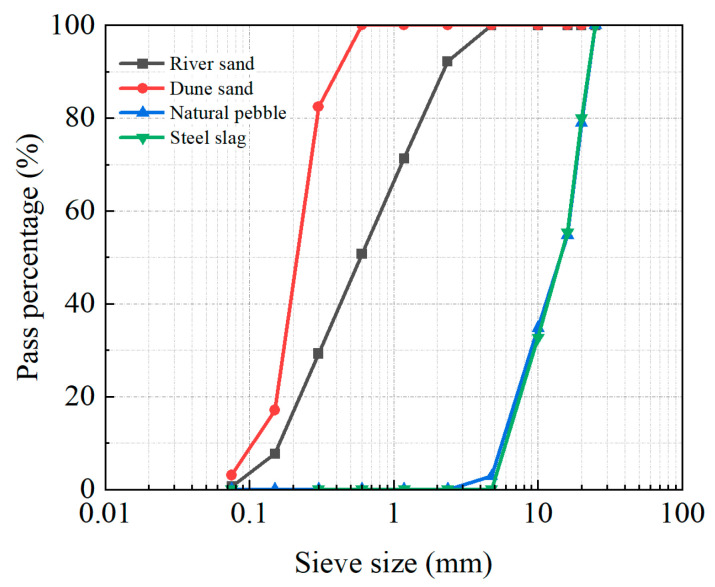
Grading curves of aggregates.

The properties of dune sand and river sand are given in Table 3. The particle grading of aggregates is plotted in Figure 1. The main difference between dune sand and river sand is that the fineness modulus of dune sand is twice that of river sand, but the average particle size of the former is half that of the latter.

A naphthalene-based high-range water reducer (HRWR) was employed, providing a water reduction rate of 25%.

The PVA fibers were purchased from one chemical technology in Shanghai. The physical properties are shown in Table 4.

### 2.2. Orthogonal Test Design

Orthogonal array testing can effectively reduce the required number of specimens and test times. This method achieves orthogonality by selecting representative points from the full factorial experiment. This study employed a four-factor, four-level orthogonal experimental design based on the L16(4^4^) orthogonal array. Based on relevant studies indicating optimal replacement ratios of 20% for dune sand (*R*_DS_) [35,36,37,38] and 50% for the optimal steel slag replacement ratio (*R*_SS_) [39,40], the current research adopted *R*_DS_ values of 10%, 20%, 30%, and 40%, alongside *R*_SS_ values of 30%, 40%, 50%, and 60%. The selection of PVA fiber lengths (*L*_PVA_) (3 mm, 9 mm, 12 mm, and 18 mm) and fiber content (*V*_PVA_) (0.1%, 0.2%, 0.3%, and 0.4%) referenced methodologies from Wang et al. [41] and Luo et al. [42]. As shown in Table 5, the factor levels were set as follows: *R*_DS_ at 10%, 20%, 30%, and 40%; *R*_SS_ at 30%, 40%, 50%, and 60%; *L*_PVA_ at 3 mm, 9 mm, 12 mm, and 18 mm; *V*_PVA_ at 0.1%, 0.2%, 0.3%, and 0.4%.

### 2.3. Mix Proportions

When making PVA fiber-reinforced concrete containing dune sand and steel slag aggregate (DS/SS-FRC) specimens, the ready-mix method is carried out according to the calculated content of each component, and then the final dosage of each component is determined. The mix proportions of DS/SS-FRC are shown in Table 6. River sand is replaced by dune sand with weight percentages of 10%, 20%, 30%, and 40%, and natural pebble is replaced by steel slag with weight percentages of 30%, 40%, 50%, and 60%. Different lengths of PVA fibers (3 mm, 9 mm, 12 mm, 18 mm) are added directly to the concrete in different volume percentages of 0.1%, 0.2%, 0.3%, and 0.4%.

### 2.4. Casting and Testing

To address the issue of PVA fiber agglomeration, a pre-treatment was performed on the fibers prior to DS/SS-FRC preparation: the antistatic agent SN was employed as a fiber dispersant at a dosage of 0.1% by mass of cement. Specifically, the fibers were pre-treated for 2 min using a high-speed mixer operating at 285 rpm. The DS/SS-FRC preparation proceeded after this pre-treatment step. According to the requirements of China Standards GB/T 50080 [43], the mixing of DS/SS-FRC mainly includes four steps. The mixing sequence began with fine aggregates blended for 5 min. Coarse aggregates were then introduced and mixed for a further minute. Subsequently, PVA fibers were uniformly dispersed and mixed for 1 min. Finally, the total mixing water combined with the HRWR was added and thoroughly mixed for 2 min. Following the slump test, the fresh mixture was placed into 100 × 100 × 100 mm^3^ cubic molds for compressive and splitting tensile strength testing as well as into 100 × 100 × 400 mm^3^ prism molds for flexural strength testing. The filled molds were placed on a vibration table to consolidate the mixture and remove air voids. Excess DS/SS-FRC was struck off level with the mold tops using a scraper. The specimens were covered with cling film and cured at 20 ± 5 °C with ≥95% relative humidity for 24 h according to GB/T 50080 [44]. After demolding, the specimens were cured to a standard curing room until reaching the specified testing age.

Following GB/T 50081 [45], compressive (*f*_cus_), splitting tensile (*f*_tts_), and flexural strength (*f*_tl_) tests were conducted after 3, 7, 28, 60, and 90 days using a 1000 kN electro-hydraulic servo testing machine. Loading rates of 0.5 MPa/s (compression) and 0.05 MPa/s (tensile/flexural) were applied until failure. Results represent the average of six specimens per test.

## 3. Test Results and Evaluations

The results of the workability and mechanical properties tests are presented in Table 7.

Orthogonal range analysis enhances experimental efficiency by distributing multiple factors across evenly spaced test levels with minimal trial requirements. Table 8 presents the results of the range analysis.

Table 8 defines *k_ij_* as the average test result of factor “*i*” at level “*j*”. The corresponding range value *R_i_* is derived from the extreme *k_ij_* differences of factor “*i*”.

### 3.1. Workability

In the slump test, except for D_40_S_60_PL_18_V_0.4_ (*R*_DS_ = 40%, *R*_SS_ = 60%, *L*_PVA_ = 18 mm, *V*_PVA_ = 0.4%), the slump values of the rest were all higher than 120 mm, indicating that the slump met the requirements of engineering construction. In the slump test, there was no stratification or segregation, which demonstrated good cohesion. There was no bleeding in the extensibility test, which indicated good water retention.

**Figure 2:**
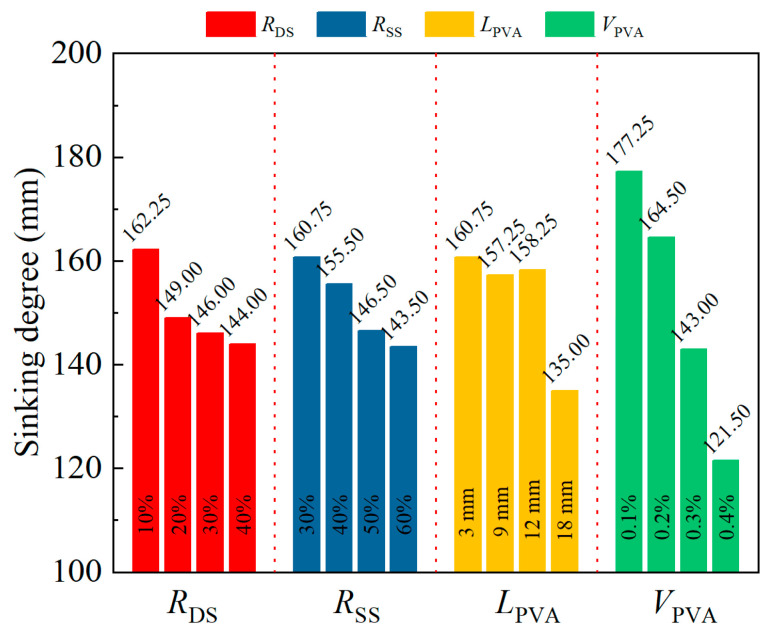
Range analysis results for the slump of specimens.

The range analysis results for slump are plotted in Figure 2. The average slump gradually decreased with an increase in *R*_DS_. As the *R*_DS_ increased from 10% to 20%, 30%, and finally to 40%, the average slump decreased from 162.25 mm to 149 mm, 146 mm, and then to 144 mm. The main reason for this decrease in slump was that compared with river sand, dune sand had more fine particles, and its average particle size was only half of that of river sand (Figure 1).

Figure 2 demonstrates the slump of DS/SS-FRC, showing a gradual decrease as the *R*_SS_ increases, which is consistent with the findings reported by Panda et al. [35]. This occurred mainly because the surface of the steel slag was porous, allowing water molecules to more easily enter and hydrate the active substances. This resulted in increased water demand, reduced moisture in the cement paste, and consequently decreased slump.

The relationship between slump and different lengths and contents of PVA fibers is also plotted in Figure 2. When the *L*_PVA_ was between 3 mm and 12 mm, changes in the average slump were not obvious. When the *L*_PVA_ increased from 12 mm to 18 mm, however, the slump decreased from 158.25 mm to 135.00 mm, showing a significant downward trend. Possible reasons are that when fiber length is within a reasonable range, its effect on concrete fluidity and water retention is not obvious. However, when fiber length is too long and exceeds a certain range, intertwining, clumping, and bunching between the fibers can occur during mixing. This adversely affects concrete fluidity, resulting in a significant decline in slump.

When the *V*_PVA_ increased from 0.1% to 0.4%, the average slump decreased from 177.25 mm to 121.50 mm, and the average slump loss exceeded 30%. This may have occurred because the PVA fiber had a strong ability to absorb free water, which increased the viscosity of the cement slurry. Additionally, the larger specific surface area of PVA fiber compared to other concrete mixtures resulted in more cement slurry surrounding the fibers, which significantly reduced concrete fluidity.

**Figure 3:**
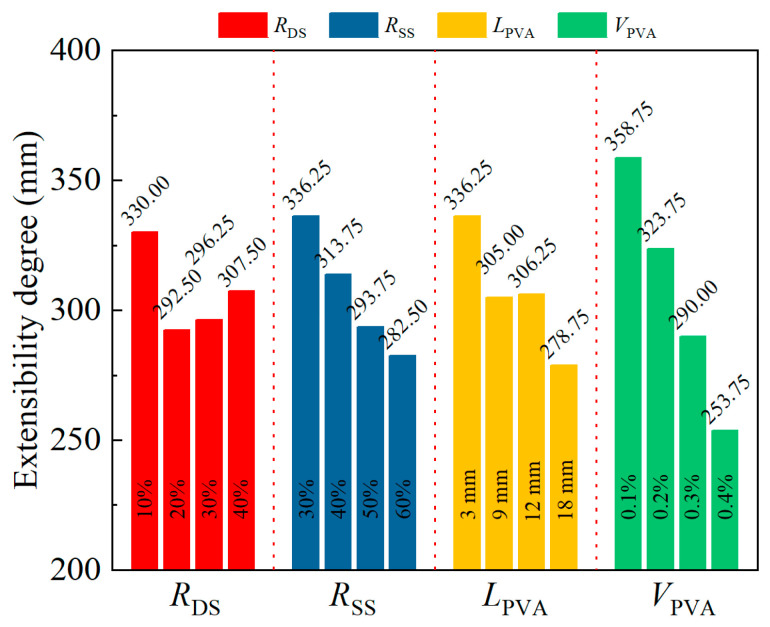
Range analysis results for the extensibility degree of specimens.

Apart from slump test results, these factors also affected extensibility. The range analysis results for extensibility are shown in Figure 3. As *R*_DS_ increased from 10% to 20%, extensibility decreased from 330.00 mm to 292.50 mm. However, extensibility gradually increased to 296.25 mm (*R*_DS_ = 30%) and 307.50 mm (*R*_DS_ = 40%) with further replacement increases.

The figure also indicates a linear decline in extensibility with increasing *R*_SS_: at 40%, 50%, and 60%, extensibility fell linearly from 336.25 mm to 313.75 mm (−6.7%), 293.75 mm (−12.6%), and 282.5 mm (−16.0%).

Additionally, extensibility dropped from 336.25 mm to 305.00 mm as fiber length increased from 3 mm to 9 mm, remained stable between 9 mm and 12 mm, and then decreased to 278.75 mm at 18 mm. Extensibility linearly decreased from 358.75 mm to 253.75 mm as PVA fiber content rose from 0.1% to 0.4%.

### 3.2. Compressive Strength

**Figure 4:**
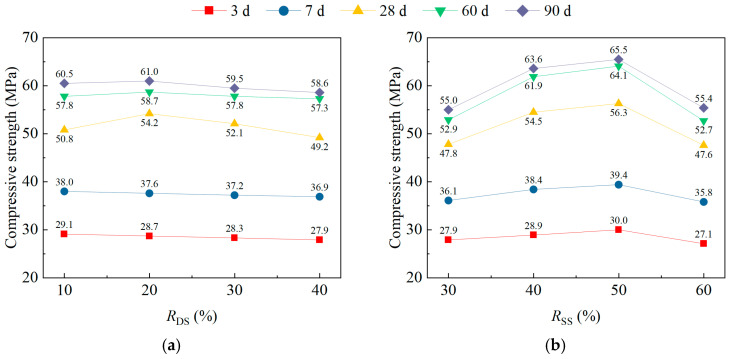

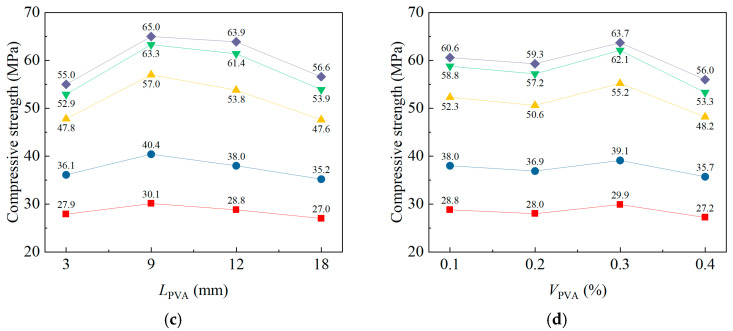
Range analysis results for the compressive strength of specimens: (**a**) *R*_DS_; (**b**) *R*_SS_; (**c**) *L*_PVA_; (**d**) *V*_PVA_.

Figure 4 illustrates the impact trend of *R*_DS_, *R*_SS_, *L*_PVA_, and *V*_PVA_ on the range analysis results for compressive strength (*f*_cus_) of DS/SS-FRC. When the *R*_DS_ kept increasing, the *f*_cus_ of DS/SS-FRC in the early strength of 3 d and 7 d decreased slightly, while the *f*_cus_ of DS/SS-FRC in the later strength of 28 d, 60 d, and 90 d increased first and then decreased (Figure 4a). With the increase in age, the influence of *R*_DS_ on the *f*_cus_ of DS/SS-FRC gradually weakens. This is mainly due to the fact that in terms of early strength, the fineness modulus of dune sand is only half that of river sand, and the grading is worse than that of river sand. At the early stage of the hydration reaction, the total surface area of the mixture increased due to the addition of dune sand, which increased the water requirement, thus affecting the hydration of concrete and ultimately slowing down the hydration reaction. The reason for the weakening influence on the later strength is that dune sand with smaller fineness grading can fully fill the small void between the coarse and fine aggregate mixture, increase the mixture compactness, and improve the *f*_cus_. The best *R*_DS_ was about 20%, and the *f*_cus_ reached its peak at 28 d, 60 d, and 90 d. Similar results were obtained by Benabed et al. [36], Menadi et al. [37], and Liu et al. [38].

From Figure 4b, with the increase in *R*_SS_, the *f*_cus_ of DS/SS-FRC at each age increased first and then decreased. They reached the peak value when the replacement rate was 50% (with a 21.2% improvement in 28 d *f*_cus_). This aligns with the findings of Saxena and Tembhurkar [40] and Fu et al. [41]. The reason is the surface of steel slag is rough and porous, which can be better combined with cementing materials than pebbles, and it enhances the interface bonding force of the coarse aggregate [9]. However, with increasing amounts of steel slag, the coarse aggregates absorbed too much water, which made the water loss in the cementing and seriously affected the bonding between the cement and the aggregate.

As shown in Figure 4c, the *f*_cus_ initially rose then fell with an increasing *L*_PVA_, peaking at 9 mm. This occurs because very short fibers generate only weak bonding/friction with cement paste, while excessively long fibers entangle and form clumps, compromising fiber–cement paste bonding.

From Figure 4d, when *V*_PVA_ increased from 0.1% to 0.2% and then to 0.3%, the *f*_cus_ of specimens at all ages first decreased slightly then increased significantly. At 0.4% content, strength decreased markedly (max. reduction: 9.4%). As flexible fibers, PVA could not independently resist pressure but enhanced concrete compactness via embedding. Optimal embedding occurred at 0.3% additional content, maximizing strength. For fiber content > 0.3%, dispersion became worse.

### 3.3. Splitting Tensile Strength

**Figure 5:**
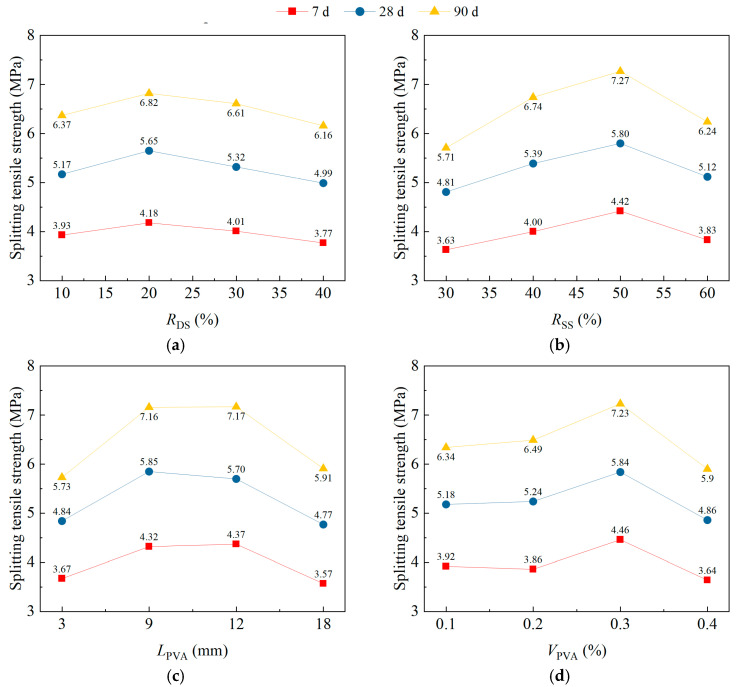
Range analysis results for the splitting tensile strength of specimens: (**a**) *R*_DS_; (**b**) *R*_SS_; (**c**) *L*_PVA_; (**d**) *V*_PVA_.

Figure 5 shows the impact trend of *R*_DS_, *R*_SS_, *L*_PVA_, and *V*_PVA_ on the range analysis results for the splitting tensile strength (*f*_tts_) of DS/SS-FRC. When the RDS was 20%, the *f*_tts_ reached its peak. When the *R*_DS_ was greater than 20%, the *f*_tts_ decreased (Figure 5a). This is because the fine aggregate of dune sand was filled in the coarse aggregate of the DS/SS-FRC, which improves the compactness of the concrete and the mechanical biting force between the aggregates, thus enhancing the *f*_tts_ of the DS/SS-FRC. When the *R*_DS_ was 20%, the compaction effect was the best.

From Figure 5b, when the *R*_SS_ increased from 30% to 60%, the *f*_tts_ at 7 d, 28 d, and 90 d first increased and then decreased. When the *R*_SS_ was 50%, the *f*_tts_ reached the peak, which is consistent with the compressive strength result.

From Figure 5c, it shows that with the increase in the *L*_PVA_, the *f*_tts_ of the specimens shows a trend of first increasing and then decreasing. When the *L*_PVA_ was 9 mm and 12 mm, the *f*_tts_ of specimen was very close and reached the maximum value.

As shown Figure 5d, the *f*_tts_ of specimen increased first and then decreased with the *V*_PVA_ increasing from 0.1% to 0.4%. The *f*_tts_ reached the maximum value as the *V*_PVA_ was 0.3%. When the fiber content is appropriate, the mechanical properties of foam concrete can be significantly improved, mainly due to the bridging effect of fibers on cracks [9]. Conversely, excessive fiber content is prone to agglomeration, thereby reducing the improvement effect on the *f*_tts_.

### 3.4. Flexural Strength

**Figure 6:**
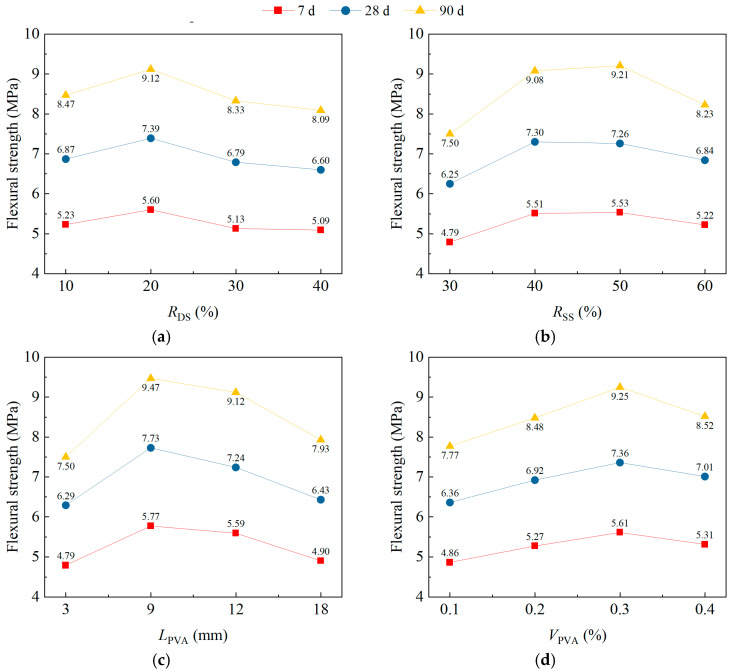
Range analysis results for the flexural strength of specimens: (**a**) *R*_DS_; (**b**) *R*_SS_; (**c**) *L*_PVA_; (**d**) *V*_PVA_.

Figure 6 shows the impact trend of *R*_DS_, *R*_SS_, *L*_PVA_, and *V*_PVA_ on the range analysis results for the flexural strength (*f*_tl_) of DS/SS-FRC. With the increase in *R*_DS_, the *f*_tl_ of the test specimen increased first and then decreased. When the *R*_DS_ was 20%, the *f*_tl_ of the specimen reached the peak (Figure 6a). The *f*_tl_ increased first and then had a declining trend with the *R*_SS_ from 30% to 60% at Figure 6b. The *f*_tl_ was very close when the *R*_SS_ was 40% and 50%, and it reached the peak value. From Figure 6c, it shows that the *f*_tl_ of the specimen rises first and is then reduced with an *L*_PVA_ of 3 mm to 18 mm. The *f*_tl_ of the DS/SS-FRC reached its peak value when the *L*_PVA_ was 9 mm. When the *V*_PVA_ increased from 0.1% to 0.3%, the *f*_tl_ increased and then declined as the *V*_PVA_ increased to 0.4%, as shown in Figure 6d. The *f*_tl_ achieved the highest at a *V*_PVA_ of 0.3%.

### 3.5. Strength Conversion Calculation Formula

The conversion formula between compressive strength and splitting tensile strength of ordinary concrete in accordance with the China Standards GB50010 [46] is as follows:

(1) $$f_{\mathrm{ts}}=0.19f_{\mathrm{cu}}^{0.75}$$

where *f*_ts_ is the splitting tensile strength of ordinary concrete; *f*_cu_ is the compressive strength of ordinary concrete.

According to the calculation in Formula (1), the differences between the predicted values and the test results are large. This may be due to the adding of dune sand, steel slag, and PVA fiber in concrete, so the parameters of Formula (1) are no longer applicable and need to be modified. Therefore, the strength conversion calculation formula for DS/SS-FRC should be modified by using the experimental data.

(2) $$f_{\mathrm{tss}}=0.093f_{\mathrm{cus}}^{1.023}$$

where *f*_tss_ is the splitting tensile strength of DS/SS-FRC; *f*_cus_ is the compressive strength of DS/SS-FRC.

Similarly, through the regression analysis of the measured test results, the conversion formula of flexural strength and compressive strength is obtained as follows.

(3) $$f_{\mathrm{tl}}=0.632f_{\mathrm{cus}}^{0.605}$$

where *f*_tl_ is the flexural strength of DS/SS-FRC.

### 3.6. Compressive Strength Growth Model

In order to obtain a fitting formula between the compressive strength and age of DS/SS-FRC, four fitting methods were used to fit the compressive strength, such as logarithmic fitting, linear fitting, quadratic polynomial fitting, and exponential fitting. The fitting results are shown in Table 9.

As can be seen from Table 9, by using the above method to fit compressive strength and age, it can be found that quadratic polynomial fitting lacks regularity. The regularity of index fitting was good, but it was obviously contrary to the general development law of concrete strength in practical engineering. Linear fitting had the worst fitting effect. The logarithmic fitting device had strong regularity and was more in line with the current prediction of DS/SS-FRC strength development. This choice was fundamentally motivated by the capacity to capture the physicochemical kinetics of cement hydration reactions, which was prioritized over mere statistical goodness of fit for specific datasets. Therefore, logarithmic fitting was recommended for the fitting formula model of the late compressive strength of DS/SS-FRC based on the early and middle strength. The formula is as follows:

(4) $$f_{\mathrm{cu},d}=8.72\ln d+20.68$$

where *d* is the age of the DS/SS-FRC; *f*_cu,*d*_ is the *d*-day compressive strength of DS/SS-FRC.

Through logarithmic fitting calculations, the relative deviations between the predicted and experimentally measured compressive strengths of DS/SS-FRC at 3 d, 7 d, 28 d, 60 d, and 90 d curing ages were determined to be 2.43%, 0.54%, 2.95%, 0.70%, and 2.15%, respectively.

## 4. Conclusions

(1) Dune sand replacement at 20% maximizes concrete mechanical properties. Steel slag replacement exhibits peak strength at 50%, following an initial increase and subsequent decline.

(2) The incorporation of PVA fibers significantly enhances the splitting tensile strength and flexural strength of DS/SS-FRC. It is recommended that the selected PVA fibers not exceed 9 mm in length, with a content not exceeding 0.3%.

(3) Through the regression analysis of experimental results, the conversion formula for the compressive strength and splitting tensile strength of DS/SS-FRC is established as $f_{\mathrm{tss}}=0.093f_{\mathrm{cus}}^{1.023}$. The conversion formula for flexural strength and compressive strength is $f_{\mathrm{tl}}=0.632f_{\mathrm{cus}}^{0.605}$. The strength growth prediction model of DS/SS-FRC is obtained as $f_{\mathrm{cu},d}=9.35\ln d+18.96$.

The present study was limited to testing mechanical properties of DS/SS-FRC at up to 90 days of aging. Future research will conduct long-term performance tests on DS/SS-FRC, thus enabling a more comprehensive evaluation of the effects of dune sand and steel slag aggregates on concrete properties.

## Figures and Tables

**Table 1:** Properties of ordinary Portland cement.

Cement Blaine (m^2^/kg)	Water Demand (%)	Setting Time (min.)		3 d Strength (MPa)		28 d Strength (MPa)	
		Initial Set	Final Set	Flexural Strength	Compressive Strength	Flexural Strength	Compressive Strength
320	27.8	150	210	6.8	22	8.8	52.7

**Table 2:** Properties of coarse aggregates.

Type	Maximum Particle Size (mm)	Apparent Density (kg/m^3^)	Bulk Density (kg/m^3^)	Crush Index (%)	Needle Particle Content (%)	Silt Content (%)
Natural pebble	20	2650	1420	8.6	9.8	0.75
Steel slag	20	3190	2240	6.3	-	0.03

**Table 3:** Properties of fine aggregates.

Sand Type	Density (g/cm^3^)		Gradation		Absorption	Average Grain Size (mm)	Silt Content (%)
	Bulk	Apparent	Fineness Modulus	Uniformity Coefficient			
Dune sand	1.56	1.44	2.125	1.44	3.84	0.218	0.23
River sand	1.43	2.92	2.876	2.92	3.28	0.600	0.01

**Table 4:** Properties of polyvinyl alcohol fiber.

Type	Equivalent Diameter (μm)	Length (mm)	Density (g/cm^3^)	Humidity Content (%)	Elongation (%)	Strength (MPa)	Elastic Modulus (GPa)
PVA fiber	15.3	3/9/12/18	1.2	<0.1	≤7	383	40

**Table 5:** The factor levels in the orthogonal test.

Level	Factor			
	*R*_DS_ (%)	*R*_SS_ (%)	*L*_PVA_ (mm)	*V*_PVA_ (%)
1	10	30	3	0.1
2	20	40	9	0.2
3	30	50	12	0.3
4	40	60	18	0.4

**Table 6:** Mix properties of the DS/SS-FRC.

Specimens ID	Influencing Factors				Mix Proportions (kg/m^3^)							
	*R*_DS_ (%)	*R*_SS_ (%)	*L*_PVA_ (mm)	*V*_PVA_ (%)	Cement	Fly Ash	River Sand	Dune Sand	Steel Slag	Natural Pebble	Water	HRWR
D_10_S_30_PL_3_V_0.1_	10	30	3	0.1	495	55	540	60	336	784	240	4
D_10_S_40_PL_9_V_0.2_	10	40	9	0.2	495	55	540	60	448	672	240	4
D_10_S_50_PL_12_V_0.3_	10	50	12	0.3	495	55	540	60	560	560	240	4
D_10_S_60_PL_18_V_0.4_	10	60	18	0.4	495	55	540	60	672	448	240	4
D_20_S_30_PL_3_V_0.3_	20	30	3	0.3	495	55	480	120	336	784	240	4
D_20_S_40_PL_9_V_0.4_	20	40	9	0.4	495	55	480	120	448	672	240	4
D_20_S_50_PL_18_V_0.1_	20	50	18	0.1	495	55	480	120	560	560	240	4
D_20_S_60_PL_12_V_0.2_	20	60	12	0.2	495	55	480	120	672	448	240	4
D_30_S_30_PL_3_V_0.4_	30	30	3	0.4	495	55	420	180	336	784	240	4
D_30_S_40_PL_18_V_0.2_	30	40	18	0.2	495	55	420	180	448	672	240	4
D_30_S_50_PL_12_V_0.3_	30	50	12	0.3	495	55	420	180	560	560	240	4
D_30_S_60_PL_9_V_0.1_	30	60	9	0.1	495	55	420	180	672	448	240	4
D_40_S_30_PL_3_V_0.2_	40	30	3	0.2	495	55	360	240	336	784	240	4
D_40_S_40_PL_12_V_0.1_	40	40	12	0.1	495	55	360	240	448	672	240	4
D_40_S_50_PL_9_V_0.3_	40	50	9	0.3	495	55	360	240	560	560	240	4
D_40_S_60_PL_18_V_0.4_	40	60	18	0.4	495	55	360	240	672	448	240	4

**Table 7:** Experimental results.

Specimens ID	Slump (mm)		Extensibility Degree (mm)		28 d *f*_cus_ (MPa)		28 d *f*_tts_ (MPa)		28 d *f*_tl_ (MPa)	
	Mean	SD	Mean	SD	Mean	SD	Mean	SD	Mean	SD
D_10_S_30_PL_3_V_0.1_	202	10.5	420	8.9	47.9	1.12	4.61	0.42	5.72	0.39
D_10_S_40_PL_9_V_0.2_	175	14.7	350	10.5	56.5	0.97	5.67	0.38	7.70	0.44
D_10_S_50_PL_12_V_0.3_	150	8.9	300	14.7	55.4	1.05	5.86	0.27	7.49	0.25
D_10_S_60_PL_18_V_0.4_	122	12.3	250	10.1	43.4	1.24	4.52	0.33	6.56	0.37
D_20_S_30_PL_3_V_0.3_	145	11.8	305	13.2	50.9	0.88	5.46	0.41	6.82	0.35
D_20_S_40_PL_9_V_0.4_	123	9.5	250	10.5	59.7	0.94	5.95	0.19	8.68	0.41
D_20_S_50_PL_18_V_0.1_	159	10.5	320	11.3	55.1	1.02	5.31	0.25	6.44	0.30
D_20_S_60_PL_12_V_0.2_	169	10.9	295	9.8	51.0	1.33	5.88	0.37	7.60	0.51
D_30_S_30_PL_3_V_0.4_	129	8.7	270	10.2	47.5	1.02	4.62	0.28	6.48	0.29
D_30_S_40_PL_18_V_0.2_	147	9.8	300	11.5	49.9	0.89	4.87	0.25	6.38	0.34
D_30_S_50_PL_12_V_0.3_	137	10.1	275	12.3	57.0	0.95	6.03	0.31	7.44	0.28
D_30_S_60_PL_9_V_0.1_	171	13.8	340	9.2	54.1	1.10	5.74	0.37	6.87	0.42
D_40_S_30_PL_3_V_0.2_	167	12.5	350	10.6	45.1	0.98	4.53	0.21	5.99	0.33
D_40_S_40_PL_12_V_0.1_	177	8.9	355	11.5	51.9	1.03	5.04	0.39	6.42	0.38
D_40_S_50_PL_9_V_0.3_	140	13.4	280	14.2	57.6	1.15	6.01	0.44	7.68	0.41
D_40_S_60_PL_18_V_0.4_	112	14.5	245	10.5	41.9	0.86	4.36	0.36	6.33	0.29

**Table 8:** The results of range analysisi.

Level	Range	Factor			
		*R*_DS_ (%)	*R*_SS_ (%)	*L*_PVA_ (mm)	*V*_PVA_ (%)
Slump (mm)	*k*_i1_	162.25	160.75	160.75	177.25
	*k_i_*_2_	149.00	155.50	157.25	164.50
	*k_i_*_3_	146.00	146.50	158.25	143.00
	*k_i_*_4_	144.00	143.50	135.00	121.50
	*R_i_*	18.25	17.25	25.75	55.75
Extensibility degree (mm)	*k*_i1_	330.00	336.25	336.25	358.75
	*k_i_*_2_	292.50	313.75	305.00	323.75
	*k_i_*_3_	296.25	293.75	306.25	290.00
	*k_i_*_4_	307.50	282.50	278.75	253.75
	*R_i_*	37.50	53.75	57.50	105.00
28 d *f*_cus_ (MPa)	*k*_i1_	50.8	47.8	47.8	52.3
	*k_i_*_2_	54.2	54.5	57.0	50.6
	*k_i_*_3_	52.1	56.3	53.8	55.2
	*k_i_*_4_	49.2	47.6	47.6	48.2
	*R_i_*	5.00	8.70	9.40	7.00
28 d *f*_tts_ (MPa)	*k*_i1_	5.17	4.81	4.84	5.18
	*k_i_*_2_	5.65	5.39	5.85	5.24
	*k_i_*_3_	5.32	5.80	5.70	5.84
	*k_i_*_4_	4.99	5.12	4.77	4.86
	*R_i_*	0.66	0.99	1.08	0.98
28 d *f*_tl_ (MPa)	*k*_i1_	6.87	6.25	6.29	6.36
	*k_i_*_2_	7.39	7.30	7.73	6.92
	*k_i_*_3_	6.79	7.26	7.24	7.36
	*k_i_*_4_	6.60	6.84	6.43	7.01
	*R_i_*	0.79	1.05	1.44	1.00

**Table 9:** Fitting formulas for compressive strength of DS/SS-FRC.

Method of Fitting	Fitting Formula	Correlation Coefficient
Logarithmic fitting	9.35ln*x* + 18.96	0.998
Linear fitting	0.33*x* + 34.64	0.805
Quadratic polynomial fitting	−0.006*x*^2^ + 0.90*x* + 28.97	0.960
Exponential fitting	24.65*x*^0.20^	0.973

## Data Availability

The original contributions presented in this study are included in the article. Further inquiries can be directed to the corresponding authors.

## References

[1] Torres A., Brandt J., Lear K., Liu J. (2017). A looming tragedy of the sand commons. Science.

[2] Cousins S. (2019). Shifting sand: Why we’re running out of aggregate. Constr. Res. Innov..

[3] Santhosh K.G., Subhani S.M., Bahurudeen A. (2021). Cleaner production of concrete by using industrial by-products as fine aggregate: A sustainable solution to excessive river sand mining. J. Build. Eng..

[4] Wang T., Zhao H.L. (2005). Fifty-year history of China desert science. J. Desert Res..

[5] Zhang T.H., Wang Q.H., Ren Q.X., Li T., Sun H.Y., Ding J.N. (2025). Compressive and flexural properties of engineered geopolymer composites incorporating dune sands. Structures.

[6] Zeng B., Zhang Z., Yang S., Mo H.J., Jin F. (2023). Alkanolamines-activated steel slag for stabilization/solidification of heavy metal contaminated soil. J. Environ. Chem. Eng..

[7] Yuan Y., Peng D.C., Shao X.Y. (2002). Crack analyzing of PVA fiber reinforced concrete beam. Ind. Constr..

[8] Gao S.L. (2006). Study on Pseudo Strain-Hardening and Fracture Characteristic of Polyvinyl Alcohol Fiber Reinforced Cementitious Composites. Ph.D. Thesis.

[9] Li T., Wang Q.H., Ren Q.X. (2024). Compressive behavior of polyvinyl alcohol engineered cementitious composites incorporating steel slag as aggregate. Constr. Build. Mater..

[10] Li T., Ren Q.X., Wang Q.H., Zhang Y.N., Ding J.N. (2024). Capillary of engineered cementitious composites using steel slag aggregate. J. Build. Eng..

[11] Zhou Z.H., Jin Q., Hu D., Zhu L., Li Z.H., Su W.Z. (2025). Long-term volume stability of steel slag sand mortar and concrete. Case Stud. Constr. Mater..

[12] Lee E., Park S.J., Kim Y.J. (2016). Drying shrinkage cracking of concrete using dune sand and crushed sand. Constr. Build. Mater..

[13] Xiong X.L., Yang Z.X., Yan X.Y., Zhang Y., Dong S., Li K., Briseghella B., Marano G.C. (2023). Mechanical properties and microstructure of engineered cementitious composites with high volume steel slag and GGBFS. Constr. Build. Mater..

[14] Yao J., Ge Y.L., Ruan W.Q., Meng J. (2024). Effects of PVA fiber on shrinkage deformation and mechanical properties of ultra-high performance concrete. Constr. Build. Mater..

[15] Guettala S., Mezghiche B. (2024). Compressive strength and hydration with age of cement pastes containing dune sand powder. Constr. Build. Mater..

[16] Park S., Lee E., Ko J., Yoo J., Kim Y. (2020). Rheological properties of concrete using dune sand. Constr. Build. Mater..

[17] Necira B., Guettala A., Guettala S. (2017). Study of the combined effect of different types of sand on the characteristics of high performance self-compacting concrete. J. Adhes. Sci. Technol..

[18] Li S.X., Qin Y.J., Cui Z., Chen C.D. (2019). Study on mix ratio of sand concrete in Taklimakan desert. New Build. Mater..

[19] Li H.P., Gui Z. (2022). Experimental study on splitting tensile strength of desert sand concrete. Fly Ash Compr. Util..

[20] Wu J.C., Shen X.D. (2017). Analysis on frost resistance and damage mechanism of aeolian sand concrete. Trans. Chin. Soc. Agric. Eng..

[21] Felekoglu B., Turkel S., Baradan B. (2007). Effect of water/cement ratio on the fresh and hardened properties of self-compacting concrete. Build. Environ..

[22] Qasrawi H., Shalabi F., Asi I. (2009). Use of low CaO unprocessed steel slag in concrete as fine aggregate. Constr. Build. Mater..

[23] Qasrawi H. (2014). The use of steel slag aggregate to enhance the mechanical properties of recycled aggregate concrete and retain the environment. Constr. Build. Mater..

[24] He X.M., Ge X.Y., Zhang B. (2023). Study on basic mechanical properties of steel slag aggregate concrete. Build. Sci..

[25] Alhozaimy A.M., Soroushian P., Mirza F. (1996). Mechanical properties of polypropylene fiber reinforced concrete and the effects of pozzolanic materials. Cem. Concr. Compos..

[26] Noushini A., Samali B., Vessalas K. (2014). Static mechanical properties of polyvinyl alcohol fibre reinforced concrete (PVA-FRC). Mag. Concr. Res..

[27] Lin H. (2006). Research on the Performance of Mechanics and Deformation of Concrete with PVA Fiber. Master’s Thesis.

[28] Qian G.F., Gao X.B., Qian C.X. (2010). Effect of PVA fiber on mechanical properties of concrete. China Concr. Cem. Prod..

[29] Hadjoudja M., Khenfer M.M., Mesbah H.A., Yahia A. (2014). Statistical models to optimize fiber-reinforced dune sand concrete. Arab. J. Sci. Eng..

[30] Ahmad S., Hakeem I., Maslehuddin M. (2016). Development of an optimum mixture of ultra-high performance concrete. Eur. J. Environ. Civ. Eng..

[31] Sun X.J., Gao Z., Cao P., Zhou C.J. (2019). Mechanical properties tests and multiscale numerical simulations for basalt fiber reinforced concrete. Constr. Build. Mater..

[32] Zhang J., Sawulet B., Li X.M. (2023). Effect of hybrid fibers on mechanical properties of concrete mixed with desert sand. Concrete.

[33] Qiao L. (2024). Research on the Mix Proportion Optimization and Mechanical and Frost Resistance Durability of Dune Sand Fiber Reinforced Concrete. Master’s Thesis.

[34] Dong Q., Wang G., Chen X., Tan J., Gu X. (2021). Recycling of steel slag aggregate in portland cement concrete: An overview. J. Clean. Prod..

[35] Panda A.P., Mohapatra B., Das S.S., Dash S., Sethy C.K. (2025). Assessment of induction furnace steel slag as a potential river sand substitute for the production of standard concrete. Struct. Concr..

[36] Benabed B., Azzouz L., Kadri E., Kenai S., Belaidi A.S.E. (2014). Effect of fine aggregate replacement with desert dune sand on fresh properties and strength of self-compacting mortars. J. Adhes. Sci. Technol..

[37] Menadi B., Kenai S., Khatib J., Ait M.A. (2009). Strength and durability of concrete incorporating crushed limestone sand. Constr. Build. Mater..

[38] Guo J.L., Yuan K., Xu J.J., Wang Y., Gan D., He M.S. (2023). The workability and mechanical performance of fly ash cenosphere–desert sand ceramsite concrete: An experimental study and analysis. Materials.

[39] Fu Q., Xue G., Xu S., Li J.J., Dong W. (2023). Mechanical performance, microstructure, and damage model of concrete containing steel slag aggregate. Struct. Concr..

[40] Saxena S., Tembhurkar A.R. (2018). Impact of use of steel slag as coarse aggregate and wastewater on fresh and hardened properties of concrete. Constr. Build. Mater..

[41] Wang J., Fu R.Z., Dong H. (2023). Carbon nanofibers and PVA fiber hybrid concrete: Abrasion and impact resistance. J. Build. Eng..

[42] Luo Z.Y., Yang X.H., Ji H.L., Zhang C.C. (2022). Carbonation model and prediction of polyvinyl alcohol fiber concrete with fiber length and content effects. Int. J. Concr. Struct. Mater..

[43] (2016). Standard for Test Method of Performance on Ordinary Fresh Concrete.

[44] (2019). Standard for Test Methods of Concrete Physical and Mechanical Properties.

[45] Liu Y.H., Li Y.Q., Jiang G.H. (2020). Orthogonal experiment on performance of mortar made with dune sand. Constr. Build. Mater..

[46] (2015). Code for Design of Concrete Structures.

